# Local Recurrence in Young Women with Breast Cancer: Breast Conserving Therapy vs. Mastectomy Alone

**DOI:** 10.3390/cancers13092150

**Published:** 2021-04-29

**Authors:** Dang Van Nguyen, Sang-Won Kim, Young-Taek Oh, O Kyu Noh, Yongsik Jung, Mison Chun, Dae Sung Yoon

**Affiliations:** 1 Department of Oncology, Hanoi Medical University, Hanoi 116001, Vietnam; drdangnguyen@gmail.com; 2 Department of Radiation Oncology, Myunggok Medical Research Institute, Konyang University College of Medicine, Daejeon 35365, Korea; swkim78@kyuh.ac.kr; 3 Department of Radiation Oncology, Ajou University School of Medicine, Suwon 16499, Korea; ohyoung@ajou.ac.kr (Y.-T.O.); okyu.noh@gmail.com (O.K.N.); 4 Department of Surgery, Ajou University School of Medicine, Suwon 16499, Korea; jungys@ajou.ac.kr; 5 Department of Surgery, Konyang University College of Medicine, Daejeon 35365, Korea

**Keywords:** breast cancer, breast conservation therapy, total mastectomy, young women, local recurrence

## Abstract

**Simple Summary:**

To date, breast conserving therapy has equivalent local control to mastectomy alone. However, it is not clear whether this finding is equally applied to young women because of the lack of large scale prospective randomized studies. In this study, we compared the local control between breast conserving therapy and mastectomy alone for young women with breast cancer. We found that young women who underwent breast conserving therapy had an approximately 2.5-fold increased risk of local recurrence compared with those receiving mastectomy alone. The prognosis of young women who had local recurrence after breast conserving therapy were poor despite the aggressive salvage treatments. Therefore, the development of more effective novel systemic treatments is required to improve treatment outcomes in young women with breast cancer receiving breast conserving therapy.

**Abstract:**

We compared the cumulative incidence of local recurrence in young patients (≤40 years) with breast cancer between breast conserving therapy (BCT) and mastectomy alone. Among 428 women with early-stage breast cancer who were treated between 2001 and 2012, 311 underwent BCT and 117 underwent mastectomy alone. Adjuvant systemic treatments were administered to 409 patients (95.6%). We compared the cumulative incidence of LR and survival rates between two groups. During a median follow-up period of 91 months, the 10-year cumulative incidence of LR was 9.3% (median interval of 36.5 months from surgery). Patients treated with BCT tended to have a higher risk for local recurrence (11.1% for BCT vs. 4.1% for mastectomy alone, *p* = 0.078). All patients with isolated LR after BCT (*n* = 23) underwent salvage mastectomy followed by systemic treatments. The 5-year distant metastasis-free survival and overall survival of patients with isolated LR after BCT were 44.2% and 82.2%, respectively. The BCT group exhibited an approximately 2.5-fold higher risk of LR than mastectomy alone group. Patients with isolated LR after BCT showed poor prognosis despite undergoing aggressive salvage treatments. The development of novel treatments should be investigated to reduce LR for improving prognosis and preserving cosmetic outcomes in young women.

## 1. Introduction

Breast conservation therapy (BCT) is the preferred primary local treatment for early-stage, invasive breast cancer because of its equivalent survival to that of mastectomy, with the additional advantage of preserving cosmetic outcomes of the involved breast [1,2].

However, performing BCT in young patients remains challenging. Since young age has been reported to be an independent risk factor for high local recurrence and poor prognosis [3,4,5], more aggressive local treatment such as mastectomy is often considered for young patients. In addition, including a small number of young patients in early randomized trials has made it difficult to confirm the efficacy and safety of BCT in young women [6,7].

Previous studies have been published to address unconfirmed legitimacy of BCT for young patients [8,9,10,11,12,13,14,15,16,17,18,19,20,21,22,23], and consistently demonstrated similar survival between BCT and mastectomy. However, the majority of these were retrospective studies and could not draw a definite conclusion. Several studies had problematic study designs, including patients who received postmastectomy radiation therapy [9,10,11,12,13,14,16,18,22,23]. In addition, only a few studies have analyzed treatment outcomes in recently treated patients [18,21,23].

As the preservation of the cosmetic outcomes of the breast is an important goal of BCT, local control should be seriously concerned before determining definitive local treatment modality. However, the comparison of local control between BCT and mastectomy has been of little interest because of similar survival. It has been questioned whether BCT also shows comparable local control with that of mastectomy because several studies have demonstrated the superiority of mastectomy in terms of local control [8,12,14,17,22,23]. Diagnosis with recurrence comes psychologically as a disaster for patients even though its extent is limited and can be salvaged by mastectomy. Furthermore, as repeated hospitalization, surgery, and adjuvant treatments are undertaken, additional medical costs and longer treatment duration can create burdens for patients.

In this study, we compared the incidence of local recurrence (LR) in young women with breast cancer between BCT and mastectomy alone. In addition, we investigated risk factors associated with increased LR among patients who underwent BCT.

## 2. Materials and Methods

The Institutional Review Board of two participating institutions approved this study with a waiver of informed consent due to its retrospective nature. All procedures performed were in accordance with the ethical standards of the institution and with the 1964 Declaration of Helsinki (and its later amendments).

We reviewed the medical records of all patients with breast cancer aged ≤40 years at diagnosis who were curatively treated between 2001 and 2012. Patients diagnosed with inflammatory breast cancer, ductal carcinoma in situ, or malignancies other than carcinoma were excluded from the analysis, as were those treated with neoadjuvant therapy or postmastectomy radiation therapy and those who refused radiation therapy after partial mastectomy.

All patients underwent definitive local treatment with either BCT or total mastectomy alone. If patients underwent total mastectomy subsequently after partial mastectomy because of a positive resection margin, local treatment was defined as mastectomy. Local treatment approaches were determined according to the surgeons’ discretion or patients’ preference. Axillary evaluation was performed with sentinel lymph node biopsy or limited axillary dissection.

Adjuvant chemotherapy was administered in 323 patients (75.5%). For patients with negative lymph nodes, the chemotherapeutic drug was either a combination of cyclophosphamide, methotrexate, and 5-fluorouracil, or an anthracycline-based regimen. Patients with positive lymph nodes received a taxane-based regimen. Adjuvant hormone therapy was administered to all patients with positive hormone receptor status for at least 5 years. In total, 406 patients (95.6%) received adjuvant systemic treatments.

Patients treated with BCT received whole-breast irradiation of 45–50 Gy with a conventional fractionation scheme, followed by a boost to the tumor bed with a median dose of 14 Gy. For patients with multiple lymph nodes, radiation was delivered conventionally to the supraclavicular area with a total dose of 45–50 Gy.

Categorical variables of clinico-pathological factors were compared between both treatment groups using the Chi-squared or Fisher’s exact test. Continuous variables, such as age and primary tumor size, were compared using the Kruskal–Wallis test. We assessed LR using cumulative incidence analysis (Gray’s test). LR was defined as the first recurrence occurring at the ipsilateral breast or chest wall. Competing risks included regional recurrence, distant metastasis, contralateral breast cancer, and intercurrent death. Competing risk regression was used to identify risk factors for LR. For patients with isolated LR after BCT, distant metastasis-free survival (DMFS) and overall survival (OS) were calculated from the date of pathologic confirmation of LR to the event of interest, using the Kaplan–Meier method. Univariate analysis was conducted using the log-rank test and multivariate analysis was conducted using Cox proportional hazard analysis. A two-sided *p* value less than 0.05 was considered statistically significant. All statistical analyses were performed with R software ver. 3.3.3.

## 3. Results

### 3.1. Patient Characteristics

We identified 428 patients who met our study criteria. Of these, 311 patients (72.7%) underwent BCT and 117 (27.3%) underwent total mastectomy alone. A comparison of patient characteristics between both treatment groups is summarized in Table 1. The median age of entire patients was 37 years (range, 19–40 years). The median tumor size was similar between both treatment groups (1.7 cm for the BCT group vs. 2.0 cm for the mastectomy alone group). Among pathological factors, human epidermal growth factor receptor 2 status and the number of positive lymph nodes were significantly different between the two treatment groups.

### 3.2. Local Recurrence

The median follow-up period was 91 months for all patients (range, 8–192 months). The median follow-up period was similar between BCT group and mastectomy group (89 months vs. 95 months, respectively). There were 30 patients (26 patients in the BCT group and 4 in the total mastectomy alone group) who experienced LR as the first recurrence, with a median interval of 36.5 months between initial local treatment and the date of pathologic confirmation. Of these, 25 patients developed isolated LR (23 patients in the BCT group and 2 patients in the total mastectomy alone group). All patients with isolated LR underwent salvage total mastectomy (BCT group) or wide excision (total mastectomy alone group).

**Figure 1:**
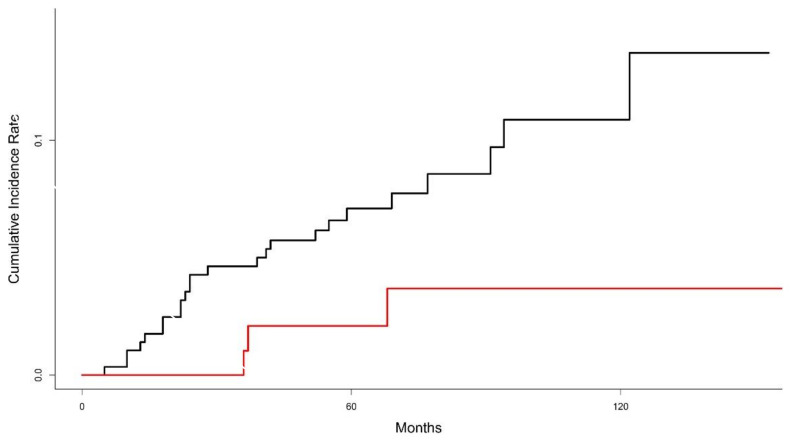
Graph of cumulative incidence of local recurrence between breast conservation therapy (black line) and mastectomy alone (red line).

The 10-year cumulative incidence of LR was 9.3% for all patients. Patients who underwent BCT showed a tendency toward higher cumulative incidence of LR, compared with those in the total mastectomy alone group (11.1% in the BCT group vs. 4.1% in the total mastectomy alone group, *p* = 0.078) (Figure 1). Multivariate competing risk regression analysis showed that BCT had a tendency toward increased risk of LR (relative risk, 3.182; 95% confidential interval, 0.0921–1.27; *p* = 0.064). Other factors, including histologic grade (*p* = 0.63), estrogen receptor (*p* = 0.80), progesterone receptor (*p* = 0.54), HER2 (*p* = 0.18), and tumor size (*p* = 0.31) were not found to be significantly associated with increased risk of LR.

We investigated to identify factors for increased risk of LR in the BCT group. On univariate analysis, all covariates including histologic grade, estrogen receptor, progesterone receptor, HER2, biological subtypes and tumor size were not significantly associated with increased the risk of LR (all *p* > 0.1).

### 3.3. Survival Rates after Local Recurrence

**Figure 2:**
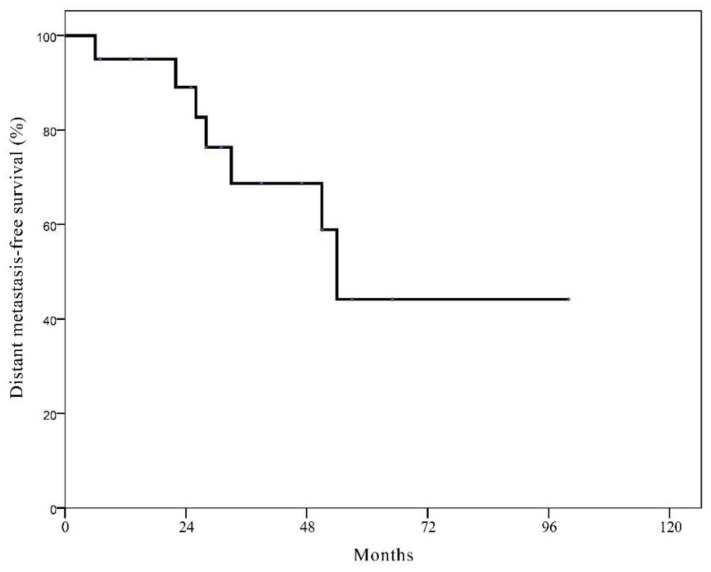
Kaplan-Meier curve of distant metastasis-free survival in patients with isolated local recurrence after breast conservation therapy.

The estimated 10-year DMFS was 84.7% for all patients. The estimated 10-year DMFS was not significantly different between two groups (84.4% in the BCT group vs. 85.7% in the mastectomy alone group, *p* = 0.533). Patients with LR showed significantly worse estimated 10-year DMFS than those without LR (47.0% vs. 88.0%; *p* < 0.001). The 10-year distant metastasis-free survival rate of patients with LR was similar between two groups (49.3% in the BCT group vs. 50.0% in the mastectomy group). For 23 patients with isolated LR after BCT, 7 patients experienced distant metastasis and the estimated 10-year DMFS rate after LR was 44.2% with a median period of 54 months (Figure 2).

**Figure 3:**
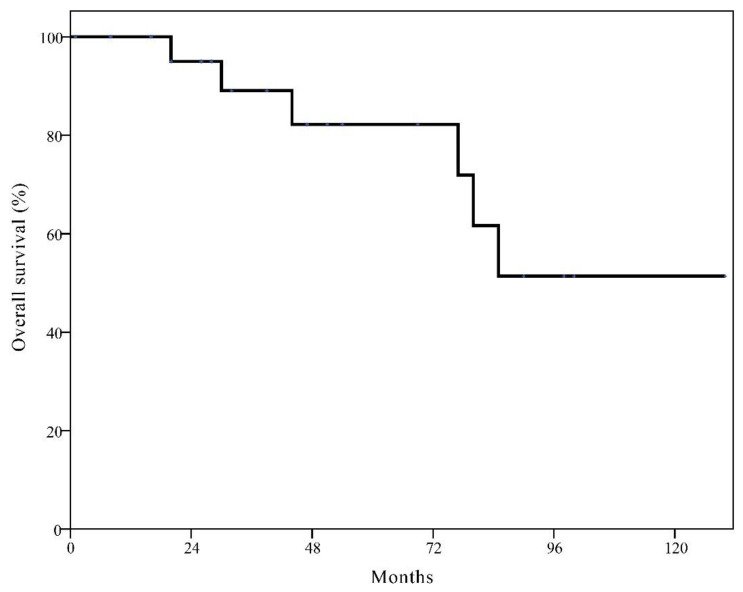
Kaplan–Meier curve of overall survival in patients with isolated local recurrence after breast conservation therapy.

The estimated 10-year OS was 89.0% for all patients. The 10-year OS was not significantly different between two groups (90.3% in the BCT group vs. 85.8% in the mastectomy alone group, *p* = 0.433). Patients with LR showed significantly worse estimated 10-year OS than those without LR (66.5% vs. 91.1%; *p* < 0.001). For patients with isolated LR after BCT, the estimated 10-year OS was 51.4% (Figure 3).

## 4. Discussion

Previously, the notion that breast cancer arising in young age presents more aggressive pathological features with advanced stage and young age is associated with increased risk of recurrence acted as barriers to perform BCT in young patients [3,4,5]. However, this concern may be unwarranted as previous studies have reported consistently equivalent survival between BCT and mastectomy [8,9,10,11,12,13,14,15,16,17,18,19,21,22,23]. The treatment outcomes of young patients undergoing BCT have also improved, owing to better preoperative imaging workups and advances in adjuvant treatments [18,24,25]. Based on this evidence, BCT is currently recommended as the first option whenever suitable, even in young patients [26,27].

Despite the evidence showing equivalent survival between BCT and mastectomy, young patients with breast cancer in the United States are increasingly choosing mastectomy instead of BCT [28,29,30]. Several factors may influence this phenomenon, including living conditions that make it difficult to receive conventional radiation therapy and fear of higher risk of LR, leading to subsequent repeated surgery [19].

Patients’ fears are not unfounded because several studies have reported higher rates of LR in young patients treated with BCT [8,12,14,17,22,23]. In this study, patients in the BCT group had a 2.5-fold higher risk of LR than those in the total mastectomy alone group and all 23 patients with isolated LR after BCT eventually underwent salvage mastectomy. The higher cumulative incidence of LR in the BCT group is supported by a recent prospectively observational cohort study (10-year LR rates of 11.7% in the BCT group vs. 4.9% in the mastectomy group, *p* < 0.001) [23].

Interestingly, the cumulative incidence curve in this study indicates that the risk of LR in the BCT group increased constantly over time, whereas a plateau was reached after 6 years in the total mastectomy alone group. van der Sangen et al. also reported similar patterns of LR according to the primary local treatments in young women [12]. Due to the continuous increase of LR in the BCT group, the difference in the incidence of LR between BCT and total mastectomy alone group will increase over time.

In this study, approximately one-third of patients with isolated LR after BCT suffered from the development of distant metastasis, even though they underwent aggressive salvage treatments. Anderson et al. also reported that of 342 patients with isolated LR after BCT, 127 (37.1%) experienced distant metastases [31]. Given the poor DMFS after aggressive salvage treatments for isolated LR in the BCT group (44.2% at 5 years), more effective novel systemic treatments should be investigated. In addition, prediction of LR using an externally validated nomogram like Dutch INFLUENCE can help to early detect LR and prevent distant metastasis [32].

The constantly increased incidence of LR and secondary development of distant metastasis in the BCT group can be dealt with by identifying patients with risk factors for LR. Therefore, we investigated risk factors associated with increased risk of LR in the BCT group. However, no clinical or pathological factors were significantly associated with increased LR. Previously, a few researchers investigated to identify risk factors associated with LR after BCT and they did not find any relevant factors [12,24,25]. This suggests that decisions for definitive local treatment approaches should not be solely based on clinical and/or pathological factors in young women. Further studies are warranted to examine risk factors at the molecular level.

This study had several limitations including inherent biases due to its retrospective design. The local treatment approach was determined according to the surgeons’ discretion or patients’ preference. Therefore, the distribution of some factors was not balanced. Although we adjusted for all available clinical and pathological factors, other unknown confounders might influence treatment outcomes. In addition, this study analyzes data from two institutions, and it is difficult to generalize the results. However, all the details of local and systemic treatments were performed based on standard procedures. During the study period, the test for BRCA mutation was not routinely performed at two institutions. Thus, this study could not evaluate the effect of BRCA mutation. Finally, there were a limited number of patients receiving adjuvant trastuzumab because it was not reimbursed for most of study period in Korea.

## 5. Conclusions

Patients in the BCT group exhibited approximately a 2.5-fold increased risk of LR compared with those in the mastectomy alone group. The incidence of LR increases continuously in the BCT group in contrast to the mastectomy alone group. Furthermore, one-third of patients with an isolated LR after BCT experienced distant metastasis despite of aggressive salvage mastectomy followed by systemic treatments. Although BCT had equivalent OS to total mastectomy alone and it can be recognized as the first local treatment option for young women with breast cancer, countermeasures are required to improve quality of life in patients treated with BCT through preservation of breast cosmetic outcomes. To reduce the risk of distant metastasis and to improve prognosis in patients with isolated LR after BCT, more effective systemic treatments should be investigated. Ultimately, it is more important to reduce the incidence of LR by developing novel treatments for improved prognosis, as well as preserving cosmetic outcomes in young women undergoing BCT.

## Figures and Tables

**Table 1:** Patient characteristics.

Variables	BCT (*n* = 311)	Mastectomy (*n* = 117)	*p* Value
Age at diagnosis (years) Median IQR	36 33 to 39	37 34 to 39	0.041
Laterality Left Right	169 (54.3%) 142 (45.7%)	54 (46.2%) 63 (53.8%)	0.161
Histologic grade Low to intermediate High	136 (46.7%) 155 (53.3%)	53 (50.5%) 52 (49.5%)	0.587
Estrogen receptor Negative Positive	104 (33.4%) 207 (66.6%)	31 (26.7%) 85 (73.3%)	0.226
Progesterone receptor Negative Positive	100 (32.2%) 211 (67.8%)	36 (31.0%) 80 (69.0%)	0.917
HER2 Negative Positive	258 (83.2%) 52 (16.8%)	71 (61.7%) 44 (38.3%)	<0.001
Intrinsic subtype Luminal A Luminal B HER2 Triple negative	190 (61.3%) 36 (11.6%) 16 (5.2%) 68 (21.9%)	63 (54.8%) 27 (23.5%) 17 (14.8%) 8 (7.0%)	<0.001
Tumor size ≤2 cm >2 cm	207 (66.6%) 104 (33.4%)	68 (58.1%) 49 (41.9%)	0.131
No. of positive LNs 0 1–3	247 (79.4%) 64 (20.6%)	74 (63.2%) 43 (36.8%)	0.001
AJCC stage I II	176 (56.6%) 135 (43.4%)	49 (41.9%) 68 (58.1%)	0.009
Adjuvant chemotherapy None CMF Anthracycline-based regimen Taxane-based regimen	75 (24.1%) 18 (5.8%) 159 (51.1%) 59 (19.0%)	30 (25.6%) 4 (3.4%) 45 (38.5%) 38 (32.5%)	0.013
Adjuvant endocrine therapy No oral regimen With LHRH analogues	92 (29.6%) 134 (43.1%) 85 (27.3%)	35 (29.9%) 56 (47.9%) 26 (22.2%)	0.523
Adjuvant trastuzumab Not indicated No Yes	259 (83.3%) 46 (14.8%) 6 (1.9%)	73 (62.4%) 39 (33.3%) 5 (4.3%)	<0.001

CMF, combination of cyclophosphamide, methotrexate and 5-fluorouracil; LHRH, luteinizing hormone-releasing hormone.

## Data Availability

The data presented in this study are available on request from the corresponding author. The data are not publicly available due to privacy and ethical restrictions.

## References

[1] Early Breast Cancer Trialists’ Collaborative Group (1995). Effects of Radiotherapy and Surgery in Early Breast Cancer—An Overview of the Randomized Trials. N. Engl. J. Med..

[2] Early Breast Cancer Trialists’ Collaborative Group (2000). Favourable and Unfavourable Effects on Long-Term Survival of Radiotherapy for Early Breast Cancer: An Overview of the Randomised Trials. Lancet.

[3] Elkhuizen P.H., Van De Vijver M.J., Hermans J., Zonderland H.M., Van De Velde C.J., Leer J.W. (1998). Local Recurrence after Breast-Conserving Therapy for Invasive Breast Cancer: High Incidence in Young Patients and Association with Poor Survival. Int. J. Radiat. Oncol. Biol. Phys..

[4] Vrieling C., Collette L., Fourquet A., Hoogenraad W., Horiot J.-C., Jager J., Oei S.B., Peterse H., Pierart M., Poortmans P. (2003). Can Patient-, Treatment- and Pathology-Related Characteristics Explain the High Local Recurrence Rate Following Breast-Conserving Therapy in Young Patients?. Eur. J. Cancer.

[5] Anders C.K., Hsu D.S., Broadwater G., Acharya C.R., Foekens J.A., Zhang Y., Wang Y., Marcom P.K., Marks J.R., Febbo P.G. (2008). Young Age at Diagnosis Correlates with Worse Prognosis and Defines a Subset of Breast Cancers with Shared Patterns of Gene Expression. J. Clin. Oncol..

[6] Fisher B., Anderson S., Bryant J., Margolese R.G., Deutsch M., Fisher E.R., Jeong J.-H., Wolmark N. (2002). Twenty-Year Follow-up of a Randomized Trial Comparing Total Mastectomy, Lumpectomy, and Lumpectomy Plus Irradiation for the Treatment of Invasive Breast Cancer. N. Engl. J. Med..

[7] Veronesi U., Cascinelli N., Mariani L., Greco M., Saccozzi R., Luini A., Aguilar M., Marubini E. (2002). Twenty-Year Follow-up of a Randomized Study Comparing Breast-Conserving Surgery with Radical Mastectomy for Early Breast Cancer. N. Engl. J. Med..

[8] Kroman N., Holtveg H., Wohlfahrt J., Jensen M.-B., Mouridsen H.T., Blichert-Toft M., Melbye M. (2004). Effect of Breast-Conserving Therapy Versus Radical Mastectomy on Prognosis for Young Women with Breast Carcinoma. Cancer.

[9] Coulombe G., Tyldesley S., Speers C., Paltiel C., Aquino-Parsons C., Bernstein V., Truong P.T., Keyes M., Olivotto I.A. (2007). Is Mastectomy Superior to Breast-Conserving Treatment for Young Women?. Int. J. Radiat. Oncol. Biol. Phys..

[10] Beadle B.M., Woodward W.A., Tucker S.L., Outlaw E.D., Allen P.K., Oh J.L., Strom E.A., Perkins G.H., Tereffe W., Yu T.-K. (2009). Ten-Year Recurrence Rates in Young Women with Breast Cancer by Locoregional Treatment Approach. Int. J. Radiat. Oncol. Biol. Phys..

[11] Bantema-Joppe E.J., De Munck L., Visser O., Willemse P.H., Langendijk J.A., Siesling S., Maduro J.H. (2011). Early-Stage Young Breast Cancer Patients: Impact of Local Treatment on Survival. Int. J. Radiat. Oncol. Biol. Phys..

[12] Van Der Sangen M.J.C., Van De Wiel F.M.M., Poortmans P.M.P., Tjan-Heijnen V.C.G., Nieuwenhuijzen G.A.P., Roumen R.M.H., Ernst M.F., Nolthenius-Puylaert M.C.B.J.E.T., Voogd A.C. (2011). Are Breast Conservation and Mastectomy Equally Effective in the Treatment of Young Women with Early Breast Cancer? Long-Term Results of a Population-Based Cohort of 1451 Patients Aged </= 40 Years. Breast Cancer Res. Treat..

[13] Mahmood U., Morris C., Neuner G., Koshy M., Kesmodel S., Buras R., Chumsri S., Bao T., Tkaczuk K., Feigenberg S. (2012). Similar Survival with Breast Conservation Therapy or Mastectomy in the Management of Young Women with Early-Stage Breast Cancer. Int. J. Radiat. Oncol. Biol. Phys..

[14] Bantema-Joppe E.J., Heuvel E.R.V.D., De Munck L., De Bock G.H., Smit W.G.J.M., Timmer P.R., Dolsma W.V., Jansen L., Schröder C.P., Siesling S. (2013). Impact of Primary Local Treatment on the Development of Distant Metastases or Death through Locoregional Recurrence in Young Breast Cancer Patients. Breast Cancer Res. Treat..

[15] Jeon Y.W., Choi J.E., Park H.K., Kim K.S., Lee J.Y., Suh Y.J. (2013). Impact of Local Surgical Treatment on Survival in Young Women with T1 Breast Cancer: Long-Term Results of a Population-Based Cohort. Breast Cancer Res. Treat..

[16] Cao J.Q., Truong P.T., Olivotto I.A., Olson R., Coulombe G., Keyes M., Weir L., Gelmon K., Bernstein V., Woods R. (2014). Should Women Younger Than 40 Years of Age with Invasive Breast Cancer Have a Mastectomy? 15-Year Outcomes in a Population-Based Cohort. Int. J. Radiat. Oncol. Biol. Phys..

[17] Xie Z., Wang X., Lin H., Wei W., Liu P., Xiao X., Xie X., Guan X., Yang M., Tang J. (2014). Breast-Conserving Therapy: A Viable Option for Young Women with Early Breast Cancer--Evidence from a Prospective Study. Ann. Surg. Oncol..

[18] Frandsen J., Ly D., Cannon G., Suneja G., Matsen C., Gaffney D.K., Wright M., Kokeny K.E., Poppe M.M. (2015). In the Modern Treatment Era, Is Breast Conservation Equivalent to Mastectomy in Women Younger Than 40 Years of Age? A Multi-Institution Study. Int. J. Radiat. Oncol. Biol. Phys..

[19] Ye J.C., Yan W., Christos P.J., Nori D., Ravi A. (2015). Equivalent Survival with Mastectomy or Breast-Conserving Surgery Plus Radiation in Young Women Aged <40 Years with Early-Stage Breast Cancer: A National Registry-Based Stage-by-Stage Comparison. Clin. Breast Cancer.

[20] Vila J., Gandini S., Gentilini O. (2015). Overall Survival According to Type of Surgery in Young (</=40 Years) Early Breast Cancer Patients: A Systematic Meta-Analysis Comparing Breast-Conserving Surgery Versus Mastectomy. Breast.

[21] Plichta J.K., Rai U., Tang R., Coopey S.B., Buckley J.M., Gadd M.A., Specht M.C., Hughes K.S., Taghian A.G., Smith B.L. (2016). Factors Associated with Recurrence Rates and Long-Term Survival in Women Diagnosed with Breast Cancer Ages 40 and Younger. Ann. Surg. Oncol..

[22] Quan M.L., Paszat L.F., Fernandes K.A., Sutradhar R., McCready D.R., Rakovitch E., Warner E., Wright F.C., Hodgson N., Brackstone M. (2017). The Effect of Surgery Type on Survival and Recurrence in Very Young Women with Breast Cancer. J. Surg. Oncol..

[23] Maishman T., Cutress R.I., Hernandez A., Gerty S., Copson E.R., Durcan L., Eccles D.M. (2017). Local Recurrence and Breast Oncological Surgery in Young Women with Breast Cancer: The Posh Observational Cohort Study. Ann. Surg..

[24] Van Laar C., van der Sangen M., Poortmans P., Nieuwenhuijzen G., Roukema J., Roumen R., Tjan-Heijnen V., Voogd A. (2013). Local Recurrence Following Breast-Conserving Treatment in Women Aged 40 Years or Younger: Trends in Risk and the Impact on Prognosis in a Population-Based Cohort of 1143 Patients. Eur. J. Cancer.

[25] Botteri E., Veronesi P., Vila J., Rotmensz N., Galimberti V., Thomazini M.V., Viale G., Orecchia R., Goldhirsch A., Gentilini O. (2017). Improved Prognosis of Young Patients with Breast Cancer Undergoing Breast-Conserving Surgery. Br. J. Surg..

[26] Cardoso F., Loibl S., Pagani O., Graziottin A., Panizza P., Martincich L., Gentilini O., Peccatori F., Fourquet A., Delaloge S. (2012). The European Society of Breast Cancer Specialists Recommendations for the Management of Young Women with Breast Cancer. Eur. J. Cancer.

[27] Partridge A.H., Pagani O., Abulkhair O., Aebi S., Amant F., Azim H.A., Costa A., Delaloge S., Freilich G., Gentilini O.D. (2014). First International Consensus Guidelines for Breast Cancer in Young Women (Bcy1). Breast.

[28] Kurian A.W., Lichtensztajn D.Y., Keegan T.H.M., Nelson D.O., Clarke C.A., Gomez S.L. (2014). Use of and Mortality after Bilateral Mastectomy Compared with Other Surgical Treatments for Breast Cancer in California, 1998–2011. JAMA.

[29] Pesce C.E., Liederbach E., Czechura T., Winchester D.J., Yao K. (2014). Changing Surgical Trends in Young Patients with Early Stage Breast Cancer, 2003 to 2010: A Report from the National Cancer Data Base. J. Am. Coll. Surg..

[30] Rutter C.E., Park H.S., Killelea B.K., Evans S.B. (2015). Growing Use of Mastectomy for Ductal Carcinoma-in Situ of the Breast among Young Women in the United States. Ann. Surg. Oncol..

[31] Anderson S.J., Wapnir I., Dignam J.J., Fisher B., Mamounas E.P., Jeong J.-H., Geyer C.E., Wickerham D.L., Costantino J.P., Wolmark N. (2009). Prognosis after Ipsilateral Breast Tumor Recurrence and Locoregional Recurrences in Patients Treated by Breast-Conserving Therapy in Five National Surgical Adjuvant Breast and Bowel Project Protocols of Node-Negative Breast Cancer. J. Clin. Oncol..

[32] Voelkel V., Draeger T., Groothuis-Oudshoorn C.G.M., De Munck L., Hueting T., Gerken M., Klinkhammer-Schalke M., Lavric M., Siesling S. (2019). Predicting the Risk of Locoregional Recurrence after Early Breast Cancer: An External Validation of the Dutch Influence-Nomogram with Clinical Cancer Registry Data from Germany. J. Cancer Res. Clin. Oncol..

